# Cytotoxicity and Antineoplastic Activities of Alkylamines and Their Borane Derivatives

**DOI:** 10.1155/MBD.1996.101

**Published:** 1996

**Authors:** Iris H. Hall, Elaine Y. Tse, Rosallah A. Muhammad

**Affiliations:** 1 Division of Medicinal Chemistry and Natural Products School of Pharmacy University of North Carolina Chapel Hill NC 27599-7360 USA; 2 Pharmacy B.S. Honors Student USA; 3 Summer Research Apprenticeship for Minority Students Program, Mathematics and Science Education Network Pre-College Program, CB#3500, 309 Peabody Hall –Babes-Bolyai– University Chapel Hill NC 27599-3500 USA

## Abstract

The alkylamines and their related boron derivatives demonstrated potent cytotoxicity against the growth of murine and human tissue cultured cells. These agents did not necessarily require the boron atom to possess potent cytotoxic action in certain tumor lines. Their ability to suppress tumor cell growth was based on their inhibition of DNA and protein syntheses. DNA synthesis was reduced because purine synthesis was blocked at the enzyme site of IMP dehydrogenase by the agents. In addition ribonucleotide reductase and nucleoside kinase activities were reduced by the agents which would account for the reduced d[NTP] pools. The DNA template or molecule may be a target of the drugs with regard to binding of the drug to nucleoside bases or intercalaction of the drug between DNA base pairs. Only some Of the agents caused DNA fragmentation with reduced DNA viscosity. These effects would contribute to overall cell death afforded by the agents.


### CYTOTOXICITY AND ANTINEOPLASTIC ACTIVITIES OF ALKYLAMINES AND THEIR BORANE DERIVATIVES

Iris H. Hall*[1], Elaine Y. Tse[1,2], and Rosallah A. Muhammad[3]

[1] Division of Medicinal Chemistry and Natural Products, School of Pharmacy, University of North Carolina, Chapel Hill, NC 27599-7360, USA
[2] Pharmacy B.S. Honors Student
[3] Summer Research Apprenticeship for Minority Students Program, Mathematics and Science Education Network Pre-College Program, CB#3500, 309 Peabody Hall, Chapel Hill, NC 27599-3500, USA

**Abstract**

The alkylamines and their related boron derivatives demonstrated potent cytotoxicity against the growth of murine and human tissue cultured cells. These agents did not necessarily require the boron atom to possess potent cytotoxic action in certain tumor lines. Their ability to suppress tumor cell growth was based on their inhibition of DNA and protein syntheses. DNA synthesis was reduced because purine synthesis was blocked at the enzyme site of IMP dehydrogenase by the agents. In addition ribonucleotide reductase and nucleoside kinase activities were reduced by the agents which would account for the reduced d[NTP] pools. The DNA template or molecule may be a target of the drugs with regard to binding of the drug to nucleoside bases or intercalaction of the drug between DNA base pairs. Only some of the agents caused DNA fragmentation with reduced DNA viscosity. These effects would contribute to overall cell death afforded by the agents.

**Introduction**

Alkylamines and their borane derivatives have shown potent hypolipidemic activity in mice, reducing LDL cholesterol and serum cholesterol and triglyceride levels while elevating HDL cholesterol levels[1,2]. The long chain derivatives, e.g. N.N-dimethyloctadecylamine borane, afforded the most potent hypolipidemic activity [2]. A positive correlation between hypolipidemic activity and cytotoxicity has previously been demonstrated for a number of boron containing compounds including amino acids, amides, esters, and peptides [3-7]. Thus, the purpose of this study is to determine if this correlation exists for a series of alkylamines and their borane derivatives. Antineoplastic and cytotoxic activity will be determined against murine L-1210 lymphoid leukemia, human Tmolt$_3$ leukemia, HeLa-S$^3$ uterine carcinoma, A549 lung, colorectal adenocarcinoma, KB nasopharynx, osteosarcoma, and glioma. The mode of action of four representative compounds in the series will be investigated for their effects on L-1210 lymphoid leukemia cell nucleic acid metabolism.

### Materials and Methods

Source of Compounds
All of the alkylamines and their boron derivatives have been synthesized previously[2] All chemical and physical characteristic of the compounds were identical to those reported [1,2]. All other chemicals, substrates and co-factors were obtained from Sigma Chemical Co. Isotopes were purchased from New England Nuclear [DuPont]

Antineoplastic Activity
Compounds **1-22** were tested for in vivo anti-neoplastic activity in the Ehrlich ascites carcinoma screen in $CF_1$ male mice (~28g) at 8 mg/kg/day, I.P. Drugs were administered for nine days. The tumor was then harvested and the volume and astrocrit were determined in order to calculate the percent inhibition of tumor growth on day 10. 6-Meracaptopurine was used as the internal standard.[5]

Cytotoxic Activity
Compound **1-22** were tested for cytotoxic activity by preparing 1 mM solutions of each drug in 0.05% Tween 80/$H_2O$ by homogenization. The solutions were sterilized by passing them through an Acrodisc filter (45μ). The following cell lines were maintained by the literature techniques [4] for murine L-1210 lymphoid leukemia, rat UMR-106 osteosarcoma, human $Tmolt_3$ acute lymphoblastic T-cell leukemia, colorectal adenocarcinoma SW480, lung A549, osteosarcoma TE418, KB epidermoid nasopharynx and HeLa-$S^3$ suspended cervical carcinoma. The protocol of Geran et al. [8] was used to assess cytotoxicity by the trypan blue exclusion for the suspended tumor cells. Solid tumor cytotoxicity was determined by the method of Leibovvitz et al. [9] using 0.2% crystal violet/20% MeOH staining and evaluation at 580 nm in a microplate reader. Standards were determined in each cell line. The compound's ED-50 values, i.e. the concentration which inhibited 50% growth, was expressed in μg/ml.

Incorporation Studies
Incorporation of labeled precursors into $^3H$-DNA, $^3H$-RNA and $^3H$-protein into $10^6$ L-1210 cells was determined by the method of Liao et al. [10]. The concentration response of compounds **4**, **12**, **14** and **22** for inhibition of DNA, RNA and protein syntheses was determined after 60 min at 25, 50 and 100 μM. 1-$^{14}C$-Glycine (53.0 mCi/mol) incorporation into purines was determined by the method of Cadman et al. [11]. $^{14}C$-Formate (53.0 mCi/mol) incorporation into pyrimidines was determined by the method of Christopherson et al.[12].

Enzyme Assays

Inhibition of various enzyme activities was carried out by first preparing the appropriate L-1210 cell homogenate or subcellular fraction, then adding the test drug during the enzyme assay. For the concentration response studies, inhibition of enzyme activity was determined at 25, 50 and 100 μM after incubation for 60 min. DNA polymerase α activity was determined in a cytoplasmic extract isolated by the method of Eichler et al.[13]. The polymerase assay was that of

Sawada et al.[14], with ($^3H$)-TTP. Messenger-, ribosomal- and transfer-RNA polymerase enzymes were isolated with different concentrations of ammonium sulfate (Anderson et al., [15] Hall et al.[16]) and the individual RNA polymerase activities were determined using $^3H$-UTP. Ribonucleoside reductase activity was measured with $^{14}C$-CDP [17]. The deoxyribonucleotides labeled with $^{14}C$-dCDP were separated from $^{14}C$-rCDP from the ribonucleotides by TLC on PEI plates. Thymidine, TMP and TDP kinase activities were measured with $^3H$-thymidine (58.3 mCi/mol) in the medium of Maley and Ochoa [18]. PRPP amidotransferase activity was determined by the method of Spassova et al. [19], and IMP dehydrogenase activity was determined with $^{14}C$-IMP (Amersham, Arlington Heights, IL) where XMP was separated on PEI plates (Fisher Scientific) by TLC (Becker et al.[20]). Carbamyl phosphate synthetase activity was determined by the method of Kalman et al. [21] and citrulline was determined colorimetrically (Archibald, [22]). Aspartate transcarbamylase activity was determined by the method of Kalman et al.[21] and carbamyl aspartate was determined colorimetrically [23]. Thymidylate synthetase activity was analyzed by the method of Kampf et al. [24]. The $^3H_2O$ measured was proportional to the amount of TMP formed from ($^3H$)-dUMP. Dihydrofolate reductase activity was determined by the spectrophotometric method of Ho et al.[25]. Protein was determined for all of the enzymatic assays (Lowry et al. [26]).

DNA Assays

Deoxyribonucleoside triphosphates were extracted by the method of Bagnara and Finch [27]. Deoxyribonucleoside triphosphates were determined by the method of Hunting and Henderson [28] with calf thymus DNA, E. coli DNA polymerase I, non-limiting amounts of the three deoxyribonucleoside triphosphates not being assayed, and either 0.4 mCi of ($^3H$-methyl)-dTTP or (5-$^3H$)-dCTP.

The effects of the compounds on DNA strand scission were determined by the methods of Suzuki et al. [29], Pera et al.[30], and Woynarowski et al. [31]. L-1210 lymphoid leukemia cells were incubated with 10 µCi thymidine methyl-$^3H$, 84.0 Ci/mmol and drug at 100 µM for 24 h at 37$^oC$. After harvesting the L1210 cells ($10^7$), the cells were centrifuged at 600 g x 10 min in PBS, washed and suspended in 1 ml of PBS. Lysis buffer (0.5 ml; 0.5 M NaOH, 0.02 M EDTA, 0.01% Triton X-100 and 2.5% sucrose) was layered onto a 5-20% alkaline-sucrose gradient (5 ml; 0.3 M NaOH, 0.7 KCl and 0.01 M EDTA) followed by 0.2 ml cell preparation. After incubating 2.5 hr at room temperature, the gradient was centrifuged at 12,000 rpm at 20$^oC$ for 60 min (Beckman rotor SW60). Fractions (0.2 ml) were collected from the top of the gradient, neutralized with 0.2 ml of 0.3 N HCl, and radioactivity measured. Thermal calf thymus DNA [ct-DNA] denaturation studies, UV absorption studies and DNA viscosity studies were conducted after incubating compounds **4**, **12**, **14** and **22** at 100 µM in PBS buffer pH 7.2 at 37oC for 24 hr[32].

**RESULTS**

A number of compounds were active in the Ehrlich ascites carcinoma screen compound **4** afforded 77% inhibition, compound **5** caused 86% inhibition and compound **11** caused 87% while compound **14** resulted in 76%

inhibition of tumor growth in vivo. Generally the alkylamine amines were not as potent as the boron derivatives. [Table 1].

Table 1. Structures and In Vivo Anti-neoplastic Activity of Alkylamine and Their Boron Derivatives at 8 mg/Kg/day, I.P.

| Compound # N = 6 | Name | Percent Inhibition Ehrlich Ascites Carcinoma Growth |
|---|---|---|
| 1 | N,N-Dimethylbenzylamine Borane | 67 |
| 2 | N,N-Dimethyl-n-butylamine Borane | 59 |
| 3 | N,N-Dimethyl-n-hexylamine Borane | 69 |
| 4 | N,N-Dimethyl-n-octylamine Borane | 77 |
| 5 | N,N-Dimethyl-n-decylamine Borane | 86 |
| 6 | N,N-Dimethyl-n-undecylamin Borane | 67 |
| 7 | N,N-Dimethyl-n-dodecylamin Borane | 58 |
| 8 | N,N-Dimethyl-n-tridecylamin Borane | 34 |
| 9 | N,N-Dimethyl-n-tetradecylamine Borane | 54 |
| 10 | N,N-Dimethyl-n-pentadecylamine Borane | 66 |
| 11 | N,N-Dimethyl-n-hexadecylamine Borane | 87 |
| 12 | N,N-Dimethyl-n-octadecylamine Borane | 52 |
| 13 | N,N-Dimethyl-n-octadecylamine | 43 |
| 14 | N-Methyl-n-octadecylamine | 76 |
| 15 | Decylamine | 37 |
| 16 | Undecylamine | 37 |
| 17 | Dodecylamine | 48 |
| 18 | Tridecylamine | 38 |
| 19 | Tetradecylamine | 56 |
| 20 | Hexadecylamine | 44 |
| 21 | Didecylamine | 45 |
| 22 | Octadecylamine | 35 |
| | 6MP | 99 |

In vitro cyctoxicity demonstrated that a number of the compounds afforded ED-50 values less than 2 μg/ml. In the L-1210 lymphoid leukemia screen compounds 1, 2, 7, 8, 9, 10, 13, 16, and 18 were active. In the human $Tmolt_3$ leukemia screen, compounds 4, 6, 7, 8, 13, 14, 15, 17, 18, 19, 21 and 22 were effective. The HeLa-$S^3$ uterine carcinoma growth was reduced by compounds 1, 3, 4, 5, 6, 8, 10, 11 and 16. KB nasopharynx growth was suppressed by compounds 14, 19 and 21. SW-480 colon adenocarcinoma growth was reduced by compounds 11, 12, 14, 16, 19, 20 and 21. Lung A549 growth was reduced by 7, 14, 18, 19, 21 and 22. Bone osteosarcoma growth was reduced by compounds 7, 9, 14, 17, 18, 19, 21 and 22. Brain glioma growth was suppressed by compounds 7, 9, 11, 14, 17, 18, 19, 21 and 22.[Table 2]

The mode of action study with four of the derivatives in L-1210 cells after 60 min incubation showed that DNA synthesis was inhibited 74% at 100 μM for compound 4 whereas compounds 12, 14 and 22 caused 24-54% reduction. RNA synthesis was only inhibited by compound 4 by 50% and compound 22 by 16%. RNA synthesis was elevated by compound 12 by 32% and by compound 14 by 79%. Protein synthesis was reduced 31% to 60% after 60 min. incubation with the agents. [Table 3-6]

DNA polymerase α activity was stimulated by compounds 4 and 12 by 52% and 106%, respectively but was inhibited 67% and 53% by compounds 14 and 22. mRNA polymerase activity was suppressed 18% to 23% by the compounds.

**rRNA polymerase activity was inhibited by all four compounds 10% to 34%. t-RNA polymerase activity was suppressed 2% to 28% after 60 min. incubation. Ribonucleoside reductase activity was suppressed 22% to 34% at 100 µM of the agents. Dihydrofolate reductase activity was reduced 16% by compound 4 but the activity was elevated 31%-48% by compounds 14 and 12. De novo synthesis of purine was inhibited approximately 37% to 57% by the four agents at 100 µM after 60 min incubation. However, the activity of the regulatory enzyme PRPP amido-transferase activity was**

**Table 2 Cytotoxicity of Alkylamines and N,N-Dimethylalkylamine Boranes in Murine and Human Tissue Culture Cell Lines**

N=4 ED-50 values = µg/ml

| Cp'd # | L-1210 Leukemia | $Tmolt_3$ Leukemia | $HeLa-S^3$ Uterine | KB Naso - pharynx | SW480 Colon | A549 Lung | Bone Osteo-Sarcoma | Brain Glioma |
|---|---|---|---|---|---|---|---|---|
| 1 | 1.69 | 2.00 | 1.53 | 3.48 | 7.52 | 8.04 | 5.73 | 7.71 |
| 2 | 1.19 | 2.89 | 2.18 | 7.38 | 2.33 | 6.85 | 5.61 | 6.63 |
| 3 | 3.77 | 3.90 | 1.44 | 6.84 | 4.42 | 6.14 | 6.66 | 7.42 |
| 4 | 2.57 | 1.96 | 1.62 | 5.67 | 5.82 | 3.76 | 5.14 | 7.72 |
| 5 | 3.86 | 4.85 | 1.36 | 6.72 | 3.97 | 5.97 | 5.44 | 7.21 |
| 6 | 3.57 | 1.35 | 1.78 | 4.26 | 6.35 | 4.87 | 3.10 | 4.90 |
| 7 | 1.47 | 1.35 | 2.21 | 6.07 | 7.27 | 1.22 | 1.50 | 0.63 |
| 8 | 1.47 | 1.94 | 1.53 | 4.39 | 7.52 | 7.86 | 3.75 | 6.53 |
| 9 | 1.43 | 2.29 | 2.29 | 4.06 | 7.56 | 7.69 | 1.78 | 1.79 |
| 10 | 0.95 | 3.11 | 1.87 | 3.61 | 7.27 | 7.83 | 3.28 | 7.72 |
| 11 | 2.30 | 2.09 | 1.57 | 5.53 | 1.62 | 2.52 | 2.38 | 1.58 |
| 12 | 2.57 | 2.06 | 2.31 | 3.70 | 1.68 | 4.89 | 3.75 | 2.78 |
| 13 | 1.83 | 1.71 | 2.63 | 6.11 | 3.63 | 6.25 | 4.32 | 8.32 |
| 14 | 3.12 | 1.43 | 2.89 | 1.27 | 1.31 | 1.15 | 1.09 | 0.27 |
| 15 | 3.59 | 1.74 | 2.21 | 3.76 | 7.52 | 3.97 | 4.91 | ----- |
| 16 | 1.56 | 2.43 | 1.87 | 4.13 | 1.56 | 6.70 | 3.51 | 6.73 |
| 17 | 2.48 | 1.43 | 2.47 | 2.93 | 5.09 | 2.10 | 1.84 | 1.72 |
| 18 | 1.47 | 1.47 | 2.29 | 2.05 | 4.07 | 1.06 | 1.98 | 1.15 |
| 19 | 2.57 | 0.97 | 2.73 | 1.95 | 1.31 | 0.995 | 1.54 | 1.13 |
| 20 | 2.68 | 3.54 | 2.68 | 4.16 | 1.25 | 4.07 | 5.66 | 5.49 |
| 21 | 2.39 | 0.775 | 3.14 | 1.58 | 1.16 | 1.06 | 1.59 | 1.47 |
| 22 | 2.75 | 1.87 | 2.12 | 3.25 | 4.03 | 1.01 | 1.05 | 0.84 |
| 5FU | 1.41 | 2.14 | 2.47 | 1.25 | 3.09 | 5.64 | ----- | 1.28 |
| AraC | 2.76 | 2.67 | 2.13 | 2.84 | 3.42 | 4.60 | ----- | 1.88 |
| HU* | 2.67 | 3.18 | 1.96 | 5.29 | 4.74 | 7.37 | 7.32 | 2.27 |

* Hydroxyurea

**not significantly affected by the agents with only 15% to 21% inhibition. Nevertheless, IMP dehydrogenase activity was markedly reduced by all four agents 38% to 75%. The first enzyme in the pyrmidine pathway were significantly reduced, i.e. carbamyl phosphate synthetase activity, which was suppressed 87% by compound 22 whereas the other three compounds caused 15% to 24% reduction in activity. Aspartate transcarbamylase activity was reduced 17% by compound 4 and was elevated by compounds 14 and 22. However, thymidylate synthtase activity was unaffected by the agents. Thymidine kinase activity was reduced 46% to 69%, TMP kinase activity was reduced 21% to 37% and TTP**

kinase activity was reduced 10% to 35% by the four agents at 100 μM. d[NTP] pools were reduced by the derivatives after 60 min. incubation. d[ATP] levels were reduced 16% to 21%. d[GTP] levels were reduced 22% to 30%. d[CTP] levels were reduced 32% to 52% whereas d[TTP] levels were slightly elevated from 105 to 21%.

Table 3 Effects of N,N-Dimethyl-n-octylamine Borane (4) on L-1210 Leukemia Cell Metabolism over 60 Min

N = 6

| Assay | Control | 25 μM | 50 μM | 100μM |
|---|---|---|---|---|
| | Percent of Control (X + SD) | | | |
| DNA synthesis | 100±5a | 53±4* | 42±2* | 26±2* |
| RNA synthesis | 100±6b | 103±7 | 62±6* | 50±3* |
| Protein synthesis | 100+5c | 119±5 | 76±4* | 69±4* |
| DNA polymerase alpha | 100±6d | 73±6 | 141±7* | 152±6* |
| mRNA polymerase | 100±7e | 112±7 | 81±5 | 81±4* |
| rRNA polymerase | 100±4f | 149±5 | 118±5 | 90±4 |
| tRNA polymerase | 100±7g | 91±6 | 89±5 | 77±5* |
| Ribonucleoside reductase | 100±5h | 87±5 | 75±5* | 69±4* |
| Dihydrofolate reductase | 100±5i | 94±6 | 86±5 | 84±6 |
| Purine de novo synthesis | 100±5j | 95±5 | 64±4* | 63±4* |
| PRPP amido transferase | 100±6k | 90±6 | 80±4* | 79±5* |
| IMP dehydrogenase | 100±5l | 89±6 | 78±5* | 49±3* |
| Carbamyl phosphate synthetase | 100±7m | 92±6 | 89±6 | 85±5 |
| Aspartate transcarboxylase | 100±6n | 101±7 | 99±6 | 83±6 |
| Thymidylate synthetase | 100±5o | 107±7 | 107±6 | 103±5 |
| Thymidine kinase | 100±6p | 79±6* | 35±4* | 35±4* |
| Thymidine monophosphat kinase | 100±7q | 72±5* | 63±5* | 63±5* |
| Thymidine diphosphate kinase | 100±6r | 117±7 | 83±6 | 77±5* |
| d(ATP) | 100±5s | | | 84±5 |
| d(GTP) | 100±6t | | | 78±4* |
| d(CTP) | 100±5u | | | 48±3* |
| d(TTP) | 100±4v | | | 110±5 |

Control values for $10^6$ cells/hr a = 7719 dpm; b = 1014 dpm, c = 17492 dpm, d = 9019 dpm, e = 1343 dpm, f = 325 dpm, g = 400 dpm, h = 48780 dpm, i = 0.144 D O.D. units, j = 28624 dpm, k = 0.0878 O.D. units, l = 19575 dpm, m = 0.807 mol N-carbamyl aspartate, n = 0.273 mmol citrulline, o = 77616 dpm, p = 1371 dpm, q = 1179 dpm, r = 1891 dpm, s = 17.07 pmoles, t = 13.58 pmoles, u = 33.60 pmoles, v = 31.04 pmoles. * P ≥ 0.001 Student's "t" test ct-DNA studies demonstrated that U.V. absorption from 220 nm to 340 nm was decreased with compounds **4**, **14** and **22** and thermal denaturation Tm values were affected by the presence of the agents after 24 hr. at 100 μM; the control Tm value was 85.3 °C, compound **4** was 77°C, compounds **12** and **22** were 70°C, compound **14** was 65°C. L-1210 DNA strand scission studies demonstrated that compounds **12** and **22** after 24 hr. at 100 μM caused essentially no DNA strand scission while compounds **4** and **14** did cause DNA fragmentation [Fig. 1]. ct-DNA viscosity studies demonstrated that the control value was 324 sec. Whereas after incubating with compounds **4**, **14** and **22**, the viscosity time had decreased to 302-318 sec.

Table 4 Effects of N,N-Dimethyl-n-octadecylamine Borane(12) on L-1210 Leukemia Cell Metabolism over 60 Min

| N = 6 | Percent of Control | | | |
|---|---|---|---|---|
| Assay | Control | 25µM | 50µM | 100µM |
| DNA synthesis | 100±5 | 80±5 | 50±4* | 49±4* |
| RNA synthesis | 100±6 | 114±6 | 106±5 | 132±7* |
| Protein synthesis | 100±5 | 63±4* | 56±5* | 48±4* |
| DNA polymerase alpha | 100±6 | 157±5* | 202±7* | 206±6* |
| mRNA polymerase | 100±7 | 74±5* | 72±4* | 71±5* |
| rRNA polymerase | 100±4 | 80±5 | 79±4* | 69±4* |
| tRNA polymerase | 100±7 | 96±6 | 77±6* | 72±6* |
| Ribonucleoside reuctase | 100±5 | 97±5 | 96±6 | 78±5* |
| Dihydrofolate reductase | 100±5 | 96±6 | 135±6* | 148±7* |
| Purine de novo synthesis | 100±5 | 60±5* | 62±4* | 54±3* |
| PRPP amido transferase | 100±6 | 85±6 | 79±5* | 78±6* |
| IMP dehydrogenase | 100±5 | 45±4* | 31±4* | 25±3* |
| Carbamyl phosphate synthetase | 100+7 | 105±6 | 100±7 | 83±5 |
| Aspartate transcarboxylase | 100±6 | 108±6 | 106±5 | 102±6 |
| Thymidylate synthetase | 100±5 | 108±6 | 102±5 | 94±5 |
| Tymidine kinase | 100±6 | 78±5* | 76±5* | 31±4* |
| Thymidine monophosphate kinase | 100±7 | 81±6 | 74±6* | 73±5* |
| Thymidine diphosphate kinase | 100±6 | 99±7 | 81±4 | 77±5* |
| d[ATP] | 100±5 | | | 80+4* |
| d[GTP] | 100±6 | | | 74±3* |
| d[CTP] | 100±5 | | | 63±4* |
| d[TTP] | 100±4 | | | 113±6 |

**Discussion**

The alkylamines and their boron derivatives were not as potent as other boronated amines, heterocyclic amine, peptides or nucleosides, but a few derivatives at 8 mg/kg/day did produce greater than 80% inhibition of in vivo tumor growth. The majority of the agents were cytotoxic in the suspended cell growth, i.e. L-1210, $Tmolt_3$ and HeLa-$S^3$. Selected derivatives demonstrated potent activity in the solid tumor cells cultures. The alkylamines which contain no boron moiety generally were more effective against the growth of the solid tumor cell cultures. This activity may be due to some type of deteregent activity of these agents being that they are long chain alkyl groups. Furthermore, compound **14**, N-methyl-n-octadecylamine demonstrated the widest activity against the growth of human cultured tumors cell lines.
The mode of action study demonstrated that L-1210 leukemic cell DNA and protein syntheses were inhibited from 25 to 100 µM within 60 min. The major effect of the agents appeared to be on de novo purine synthesis with the regulator enzyme site IMP dehydrogenase being blocked by the agents. The magnitude of reduction of IMP dehydrogenase

Table 5 Effects of N-methyl-n-octadecylamine(14) on L-1210 Leukemia Cell Metabolism over 60 Min

N = 6 Percent of Control

| Assay | Control | 25μM | 50μM | 100μM |
|---|---|---|---|---|
| DNA synthesis | 100±5 | 76±5* | 69±5* | 76±4* |
| RNA synthesis | 100±6 | 155±6* | 176±5* | 179±6* |
| Protein synthesis | 100+5 | 74±4 | 62±5 | 51±5* |
| DNA polymerase alpha | 100±6 | 100±5 | 53±4 | 33±3* |
| mRNA polymerase | 100±7 | 96±6 | 83±6 | 77±5* |
| rRNA polymerase | 100±4 | 78±5* | 75±5* | 68±5* |
| tRNA polymerase | 100±7 | 103±7 | 86±7 | 82±6 |
| Ribonucleoside reductase | 100±5 | 104±6 | 79±6 | 68±5* |
| Dihydrofolate reductase | 100±5 | 107±7 | 131±6 | 131±5 |
| Purine de novo synthesis | 100±5 | 57±5* | 47±5* | 43±4* |
| PRPP amido transferae | 100±6 | 87±6 | 87±5 | 82±6 |
| IMP dehydrogenase | 100±5 | 75±5* | 60+5* | 45±4* |
| Carbamyl phosphate synthetase | 100±7 | 81±6 | 76±5* | 76±4* |
| Aspartate transcarboxylase | 100±6 | 119±7 | 139+7* | 122±6 |
| Thymidylate synthetase | 100±5 | 103±5 | 108±6 | 98±6 |
| Tymidine kinase | 100±6 | 68+6 | 44±5* | 38±4* |
| Thymidine monophosphate kinase | 100±7 | 80±7 | 71±5* | 69±4* |
| Thymidine diphosphate kinase | 100±6 | 86±6 | 72±6* | 65±5* |
| d[ATP] | 100+5 | | | 82±4 |
| d[GTP] | 100±6 | | | 70±5* |
| d[CTP] | 100±5 | | | 61±5* |
| d{TTP] | 100±4 | | | 115±6* |

activity was of sufficient amount to explain the observed reduction of purine synthesis as well as DNA synthesis. Other metabolic site which were affected marginally by the agents were m-, r-and t-RNA polymerases, ribonucleoside reductase, carbamyl phosphate synthetase, and nucleoside kinases. The inhibition of the activities of these enzymes would be additive with regard to inhibiting DNA synthesis and tumor cell death. The reduction in some of the d[NTP] pools would also lower DNA synthesis. The reduction in these pools probably is due to the inhibition of purine and pyrimidine <u>de novo</u> synthetic pathways for deoxytriphosphate nucleosides as well as ribonucleoside reductase activity for the conversion of ribosenucleotides to deoxyribonucleotides. ct-DNA studies with the agents suggest that there was some type of interaction with the nucleosides of DNA as demonstrated by the decrease in U.V. absorption, decreased Tm values and lower DNA viscosity. In the L-1210 cells the observed DNA fragmentation would cause cell death and explain the reduction in DNA viscosity.

Table 6 Effects of Octadecylamine(22) on L-1210 Leukemia Cell Metabolism over 60 Min

N = 6

| Assay | Control | 25μM | 50μM | 100μM |
|---|---|---|---|---|
| | Percent of Control | | | |
| DNA synthesis | 100±5 | 90±6 | 73±5* | 46±5* |
| RNA synthesis | 100±6 | 118±6 | 115±7 | 84±5 |
| Protein synthesis | 100±5 | 86±6 | 85±5 | 40±4* |
| DNA polymerase | 100+6 | 94±5 | 81±6 | 47±5* |
| mRNA polymerase | 100±7 | 89±6 | 83±6 | 82±6 |
| rRNA polymerase | 100±4 | 99±5 | 83±6 | 66+5* |
| tRNA polymerase | 100±7 | 103±6 | 102+6 | 98±5 |
| Ribonucleoside reductase | 100±5 | 130±6 | 84±5 | 66±4* |
| Dihydrofolate reductase | 100±5 | 119±7 | 116±6 | 96±5 |
| Purine synthesis | 100±5 | 70±4* | 54±5* | 51±5* |
| PRPP amido transferase | 100±6 | 94±5 | 86±6 | 85±6 |
| IMP dehydrogenase | 100±5 | 194±7 | 119±6 | 62±5* |
| Carbamyl phosphate synthetase | 100±7 | 71±5* | 31±4* | 13±3* |
| Aspartate transcarboxylase | 100±6 | 123±5 | 159±6* | 164±6* |
| Thymidylate synthetase | 100±5 | 105±6 | 110±6 | 104±6 |
| Thymidine kinase | 100±6 | 91±5 | 74±5* | 54±5* |
| Thymidine monophosphat kinase | 100±7 | 92±6 | 76±5* | 70±5* |
| Thymidine diphosphate kinase | 100±6 | 105±7 | 101±6 | 90±5 |
| d[ATP] | 100±5 | | | 79±5* |
| d[GTP] | 100±6 | | | 76±4* |
| d[CTP] | 100±5 | | | 68±4* |
| d[TTP] | 100±4 | | | 115±6 |

**Acknowledgements:**

Compounds used in this study were supplied by U.S. Borax Research Corporation, Anaheim, CA 92801 for which we are appreciative.
Funding for this reaserch was provided by the UNC Pharmacy Foundation Seed Grant.

**References**

1. Hall IH, Griffin TS, Docks EL, Brotherton RJ, Futch G. Hypolipidemic activity of N,N-dimethylamine borane in rodents, J. Pharm. Sci. 1986, 75: 706.
2. Griffin TS, Docks EL, Brotherton RJ, Hall IH. The hypolipidemic activity of alkylamines and their borane derivatives: structure-activity relationship in rodents. Eur. J. Med. Chem. 1991, 26: 517.
3. Hall IH, Gilbert CJ, McPhail, Morse KW, Hassett K, Spielvogel BF. Antineoplastic activity of boron analogues of α-amino acids. J. Pharm. Sci. 1985, 74: 755.
4. Sood CK, Sood A, Spielvogel BF, Yousef JA, Burhmman S, Hall IH. Synthesis and anti-neoplastic activity of some cyano-, carbomethoxy- and carbamoylborane adducts of heterocycic amines, J.Pharm. Sci. 1991, 80: 1133.
5. Hall IH, Spielvogel BF, McPhail AT. Antineoplastic activity tetrakis-μ-(trimethylamine-boranecarboxylato)-bis(trimethylaminecarboxyborane)dicopper(II) in Ehrlich ascites carcinoma. J. Pharm. Sci. 1984, 73: 222.

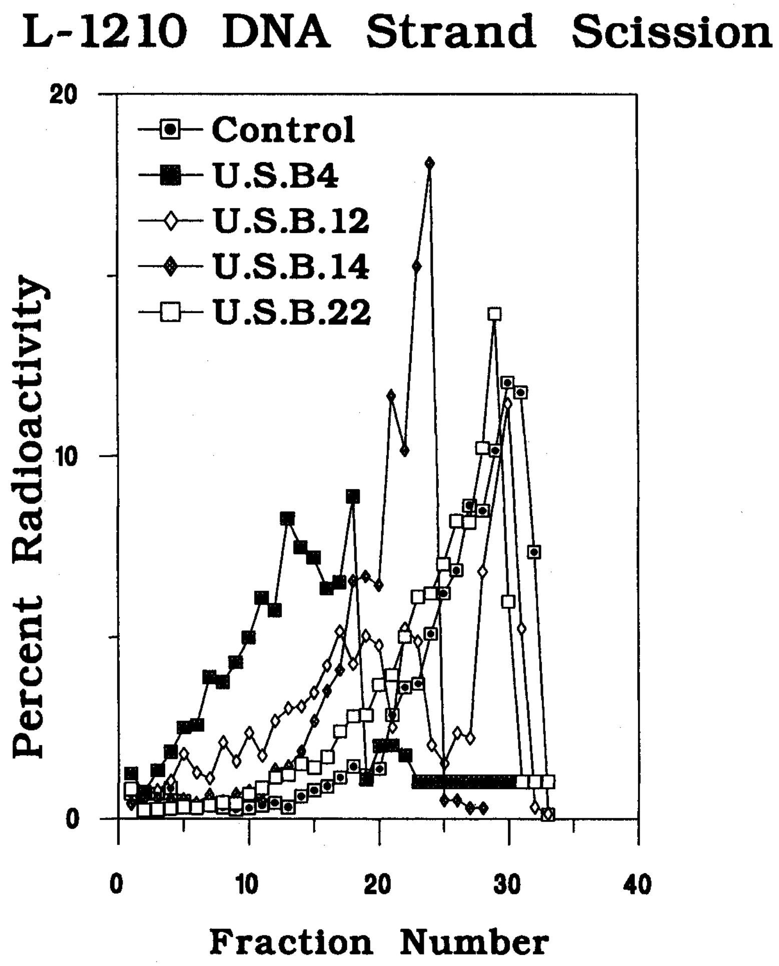

Fig. 1 Effects od Alkylamines and their Boranes on DNA Fragmentation

---

6. Sood A, Sood CK, Spielvogel BF, Hall IH. Boron amalogues of amino acids VI. Synthesis and characterization od di- and tripeptide analogues as antineoplastic, anti-inflammatory and hypolipidemic agents. Eur. J. Med. Chem. 1990, 25: 301.

7. Hall IH, Hall ES, Chi LK, Shaw BR, Sood A, Spielvogel BF. Antineoplastic activity of boron containing thymidine nucleosides in Tmolt3 leukemic cells, Anticancer Res. 1992, 12:1091.

8.Geran RJ, Greenburg NH, MacDonald MM, Schumacher AM, Abbott BJ. Protocols for screening chemical agents and natural products against animal tumors and other biological systems. Cancer Chemo Rep 1972; 3: 9.

9. Leibovitz AL, Stinson JC, McComb WB III, McCoy CE, Mazur KC, Mabry ND. Classification of human colorectal adenocarcinoma cell lines. Cancer Res 1976; 36: 4562.
10. Liao LL, Kupchan SM, Horwitz SB. Mode of action of the antitumor compound bruceatin, an inhibitor of protein synthesis. Mol Pharmacol 1976; 12: 167.
11. Cadman E, Heimer R, Benz C. The influence of methotrexate pretreatment on S-flaxorouracil metabolism in L1210 cells. J Biol Chem 1981; 256: 1695.
12. Christopherson RI, Yu ML, Jones ME. An overall radioassay for the first three reactions of de novo pyrimidine synthesis. Anal Biochem 1981; 11: 240.
13. Eichler DC, Fisher PA, Korn D. Effect of calcium on the recovery distribution of DNA polymerase from cultured human cells. J Biol Chem 1977; 252: 4011.
14. Sawada H, Tatsumi K, Sadada M, Shirakawa S, Nakamura T, Wakisaka G. Effects of neocarzinostatin on DNA synthesis in L1210 cells. Cancer Res 1974; 34: 3341.
15. Anderson KM, Mendelson IS, Guzik G. Solubilized DNA-dependent nuclear RNA polymerases from the mammary glands of late-pregnant rats. Biochem Biophys Acta 1975; 383: 56.
16. Hall IH, Carlson GL, Abernathy GS, Piantadosi C. Cycloalkanones IV. Antifertility agents. J Med Chem 1974; 17: 1253-1257.
17. Moore EC, Hurlbert RB. Regulation of mammalian deoxyribonucletide biosynthesis by nucleotide or activators and inhibitors. J Biol Chem 1966; 241: 4802.
18. Maley F, Ochoa S. Enzymatic phosphorylation of deoxycytidylic acid. J Biol Chem 1958; 233: 1538.
19. Spassova MK, Russev GC, Goovinsky EV. Some pyrazoles as inhibitors of purine biosynthesis de novo. Biochem Pharmacol 1976; 25: 923.
20. Becker JH, Lohr GW. Inosine-51-phosphate dehydrogenase activity in normal and leukemic blood cells. Klin Wochenschr 1979; 57: 1109.
21. Kalman SM, Duffield PH, Brzozwki TJ. Purification and properties of a bacterial carbamyl phosphate synthetase. J Biol Chem 1966; 241: 871.
22. Archibald RM. Determination of citrulline and allontoin and demonstration of citrulline in blood plasma. J Biol Chem 1944; 156: 121.
23. Koritz SB, Gohen PP. Colorimetic determination of carbamyl amino acid and related com pounds. J Biol Chem 1954; 209: 145.
24. Kampf A, Barfknecht RL, Schaffer PJ, Osaki S, Mertes MP. Synthetic inhibitors of Escherichia coli calf thymus and Ehrlich ascites tumor thymidylate synthetase. J Med Chem 1976; 19: 903.
25. Ho YK, Hakala T, Zakrzewski SF. 5-(1-Adamantyl) pyrimidines as inhibitors of folate metabolism. Cancer Res 1971; 32: 1023.
26. Lowry OH, Rosebrough J, Farr AL, Randall RJ. Protein measurement with folin phenol reagent. J Biol Chem 1951; 193: 265.
27. Bagnara AS, Finch LR. Quantitative extraction and estimation of intracellular nucleostide-trip hosphate in Escherichia coli. Anal Biochem 1971; 45: 24.
28. Hunting D, Henderson JF. Determination of deoxyribonucleoside triphosphates using DNA polymerase: a critical evaluation. Can J Biochem 1982; 59: 723.
29. Suzuki H, Nishimura T, Muto SK Tanaka N. Mechanism of action of macromomycin: DNA strand scission, inhibition of DNA synthesis mitosis. J Antibacteriol 1978; 32: 875.

30. Pera JF Sr, Rawlings CJ, Shackleton J, Roberts JJ. Quantitative aspects of the formation and loss of DNA. Biochem Biophy Acta 1981; 655: 152.
31. Woynarowski JW, Beerman TA, Konopa J. Introduction of deoxyribonucleic acid damage in HeLa $S^3$ cells by cytotoxic and antitumor sesquiterpine lactones. Biochem Pharmacol 1981; 30: 3005.
32.Zhao Y, Hall IH, Oswald CB, Yokoi T, Lee KH. Anti-malarial agents III. Mechanism of action of artesunate against Plasmodium berghi infection. Chem Phar Bull 1987; 35: 2052.

**Received: February 12, 1996 - Accepted: March 5, 1996 - Received in revised camera-ready format: March 21, 1996**

